# Impact of Virtual Reality on stress management and psychosocial risk prevention in Healthcare Workers: A Literature Review

**DOI:** 10.1192/j.eurpsy.2026.11849

**Published:** 2026-07-31

**Authors:** C. Sridi, I. Kacem, J. Ben Khelifa, R. Nakhli, F. Chelly, N. Belhadj, N. Gannoun, A. Fki, M. Maoua

**Affiliations:** 1 Occupational Medicine Department, Sahloul University Hospital; 2 University of Sousse, Faculty of Medicine of Sousse; 3 Research Laboratory LR19SP03; 4 Occupational Medicine Department, Farhat Hached University Hospital; 5 Clinical Psychology Unit of Sahloul University Hospital, Sousse, Tunisia

## Abstract

**Introduction:**

The healthcare sector faces significant challenges, including high occupational stress and burnout, exacerbated by global health crises. Virtual Reality (VR) technology offers a promising solution for mental health interventions, providing immersive experiences for stress alleviation and relaxation.

**Objectives:**

This review explores the impact of VR on stress management and psychosocial risk prevention among healthcare workers.

**Methods:**

This literature review synthesized evidence from studies published between 2020 and 2025, retrieved from PubMed, Web of Science, Scopus, and Google Scholar. The search focused on VR interventions for stress reduction and psychosocial risk prevention in healthcare professionals. Inclusion criteria included randomized controlled trials, quasi-experimental, and pre-post studies. Data extraction focused on intervention details, outcomes (stress, anxiety, depression), and key findings.

**Results:**

Our review indicates that virtual reality interventions effectively reduce stress and anxiety among healthcare workers. Studies show significant reductions in perceived stress and anxiety, with some demonstrating specific decreases in depression and stress. Even single VR relaxation sessions have been shown to lower perceived stress in frontline workers. While the benefits appear immediately, the long-term effects and impact on specific psychosocial risks like burnout require further investigation with larger, controlled studies.

**Conclusions:**

VR interventions show considerable promises for stress management and psychosocial risk prevention in healthcare. They offer immediate relief from stress and anxiety. Future research should focus on long-term efficacy, impact on specific risks, and comparative effectiveness with other interventions. Integrating VR into occupational health strategies could provide a scalable solution for healthcare workers’ well-being.

**Disclosure of Interest:**

None Declared

